# StackTTCA: a stacking ensemble learning-based framework for accurate and high-throughput identification of tumor T cell antigens

**DOI:** 10.1186/s12859-023-05421-x

**Published:** 2023-07-28

**Authors:** Phasit Charoenkwan, Nalini Schaduangrat, Watshara Shoombuatong

**Affiliations:** 1 Modern Management and Information Technology, College of Arts, Media and Technology, Chiang Mai University, Chiang Mai, 50200 Thailand; 2 Center for Research Innovation and Biomedical Informatics, Faculty of Medical Technology, Mahidol University, Bangkok, 10700 Thailand

**Keywords:** T-cell antigen, Bioinformatics, Stacking strategy, Feature selection, Machine learning

## Abstract

**Background:**

The identification of tumor T cell antigens (TTCAs) is crucial for providing insights into their functional mechanisms and utilizing their potential in anticancer vaccines development. In this context, TTCAs are highly promising. Meanwhile, experimental technologies for discovering and characterizing new TTCAs are expensive and time-consuming. Although many machine learning (ML)-based models have been proposed for identifying new TTCAs, there is still a need to develop a robust model that can achieve higher rates of accuracy and precision.

**Results:**

In this study, we propose a new stacking ensemble learning-based framework, termed StackTTCA, for accurate and large-scale identification of TTCAs. Firstly, we constructed 156 different baseline models by using 12 different feature encoding schemes and 13 popular ML algorithms. Secondly, these baseline models were trained and employed to create a new probabilistic feature vector. Finally, the optimal probabilistic feature vector was determined based the feature selection strategy and then used for the construction of our stacked model. Comparative benchmarking experiments indicated that StackTTCA clearly outperformed several ML classifiers and the existing methods in terms of the independent test, with an accuracy of 0.932 and Matthew's correlation coefficient of 0.866.

**Conclusions:**

In summary, the proposed stacking ensemble learning-based framework of StackTTCA could help to precisely and rapidly identify true TTCAs for follow-up experimental verification. In addition, we developed an online web server (http://2pmlab.camt.cmu.ac.th/StackTTCA) to maximize user convenience for high-throughput screening of novel TTCAs.

**Supplementary Information:**

The online version contains supplementary material available at 10.1186/s12859-023-05421-x.

## Introduction

Tumor cells generate molecules called tumor antigens (TAs). TAs are classified into two types: tumor associated antigens (TAAs) and tumor specific antigens (TSAs). TAAs are self-proteins which are highly expressed in tumor cells in comparison to normal cells, while TSAs are found solely in tumor cells [1, 2]. The human body is capable of recognizing TAs and initiating the innate and adaptive immune responses of the body to eliminate cancerous growths. Innate immune cells, (i.e., neutrophils, macrophages, NK cells, dendritic cells, and others) can quickly respond and to offer defense mechanisms that are nonspecific. The adaptive immune system, comprising T-cells and B-cells, is a more intricate and slower process to target antigens. However, it has the potential to create a robust and targeted immune response to combat tumors or cancers [3]. Dendritic cells (DCs) that present antigens break down TAs and exhibit small peptides through major histocompatibility complex class I (MHC-I) to activate CD8+ T-cells that are cytotoxic, or through MHC class II to stimulate CD4+ T-cells that are helper T-cells. However, CD8+ T-cells are crucial for eradicating tumors and performing surveillance of the immune system to target cancer cells [4, 5]. Hence, T-cell epitopes linked with TAs are one of the most important targets for developing cancer immunotherapy, which can help eliminate diseases and prevent their recurrence. In recent times, the identification of peptides originating from TAs as epitopes has been used as immunotherapeutic agents to combat various types of tumors and cancers. [3, 4, 6, 7]. For a T-cell antigen to be an ideal target in cancer immunotherapy, it needs to fulfil several criteria. These include exhibiting specificity to the tumor, which means that it should be highly expressed in cancerous tissues but should not trigger autoimmunity or immune tolerance. Additionally, the antigen should be prevalent and abundant in tumor cells, especially if it plays a crucial role in oncogenesis and can prevent the tumor from evading the immune system. Furthermore, the antigen should be immunogenic, meaning that it should be capable of generating an immune response, which can be assessed by cytokine release, tumor cytolysis and most importantly, T-cell recognition. Finally, epitopes with favorable properties, such as optimal length, hydrophobicity, and aromaticity, could be highly effective [1, 8–10]. In order to create successful experiments for personalized and precise immunotherapy, it is crucial to have a comprehensive understanding of the immunogenic epitopes found on tumor antigens.

The existence of large peptide databases, such as immune epitope database (IEDB) [11], TANTIGEN [12], and TANTIGEN 2.0 [13], is expected to aid in the identification of tumor T-cell antigens (TTCAs) that bind to MHC-I molecules. By using sequence information alone, computational methods have the potential to rapidly and precisely identify TTCAs, which can be a more time-efficient and cost-effective alternative to experimental approaches. This is especially important given the laborious and expensive nature of test-based discovery, making it imperative to develop efficient computational methods for TTCAs identification [14–16]. To date, there are a variety of computational approaches that have been created for TTCA identification based on sequence information, including TTAgP1.0 [17], iTTCA-Hybrid [18], TAP1.0 [19], iTTCA-RF [20], iTTCA‑MFF [21], and PSRTTCA [22]. Table 1 summarizes the existing computational approaches according to the applied benchmark datasets, machine learning (ML) methods, and web server availability. According to the applied ML methods, the six existing computational approaches can be categorized into two groups, i.e., single ML-based (TTAgP1.0 [17], iTTCA-Hybrid [18], TAP1.0 [19], iTTCA-RF [20], and iTTCA‑MFF [21]) and ensemble learning-based (PSRTTCA [22]) methods. Among the existing computational approaches, PSRTTCA [22] was recently developed constructed on RF-based meta-approach. In PSRTTCA, a pool of propensities for amino acids and dipeptides was estimated by using the scoring card method (SCM) and then treated as the input feature vector for the construction of a meta-predictor. More details for the existing computational approaches are summarized in two previous studies [21, 22]. Although the existing computational approaches attained reasonably good performances, their performance is still not yet satisfactory in terms of the independent test dataset. For example, PSRTTCA, which performed best among various TTCA predictors, could provide an accuracy (ACC) of 0.827 and Matthew’s correlation coefficient (MCC) of 0.654.

**Table 1:** Summary of existing computational methods for the prediction of QSPs

Methods/tools	Year	Method^a^	Type	Benchmark dataset^b^	Reliable negative dataset	Web server availability
TTAgP1.0 [17]	2019	RF	Single	JFB2019	Yes	No
iTTCA-Hybrid [18]	2018	RF	Single	PC2020	No	Yes
TAP1.0 [19]	2021	QDA	Single	JHB2021	Yes	Yes
iTTCA-RF [20]	2021	RF	Single	PC2020	No	Yes
iTTCA‑MFF [21]	2022	SVM	Single	PC2020	No	No
PSRTTCA [22]	2023	RF	Ensemble	JHB2021	Yes	Yes
StackTTCA	This study	ET	Ensemble	JHB2021	Yes	Yes

^a^*ET* Extremely randomized trees, *QDA* Quadratic discriminant analysis, *RF* Random forest, *SVM* Support vector machine

^b^The JFB2019, PC2020, and JHB2021 datasets (TTCAs, non-TTCAs) consist of (553, 369), (470, 318), and (592, 592), respectively

**Fig. 1:**
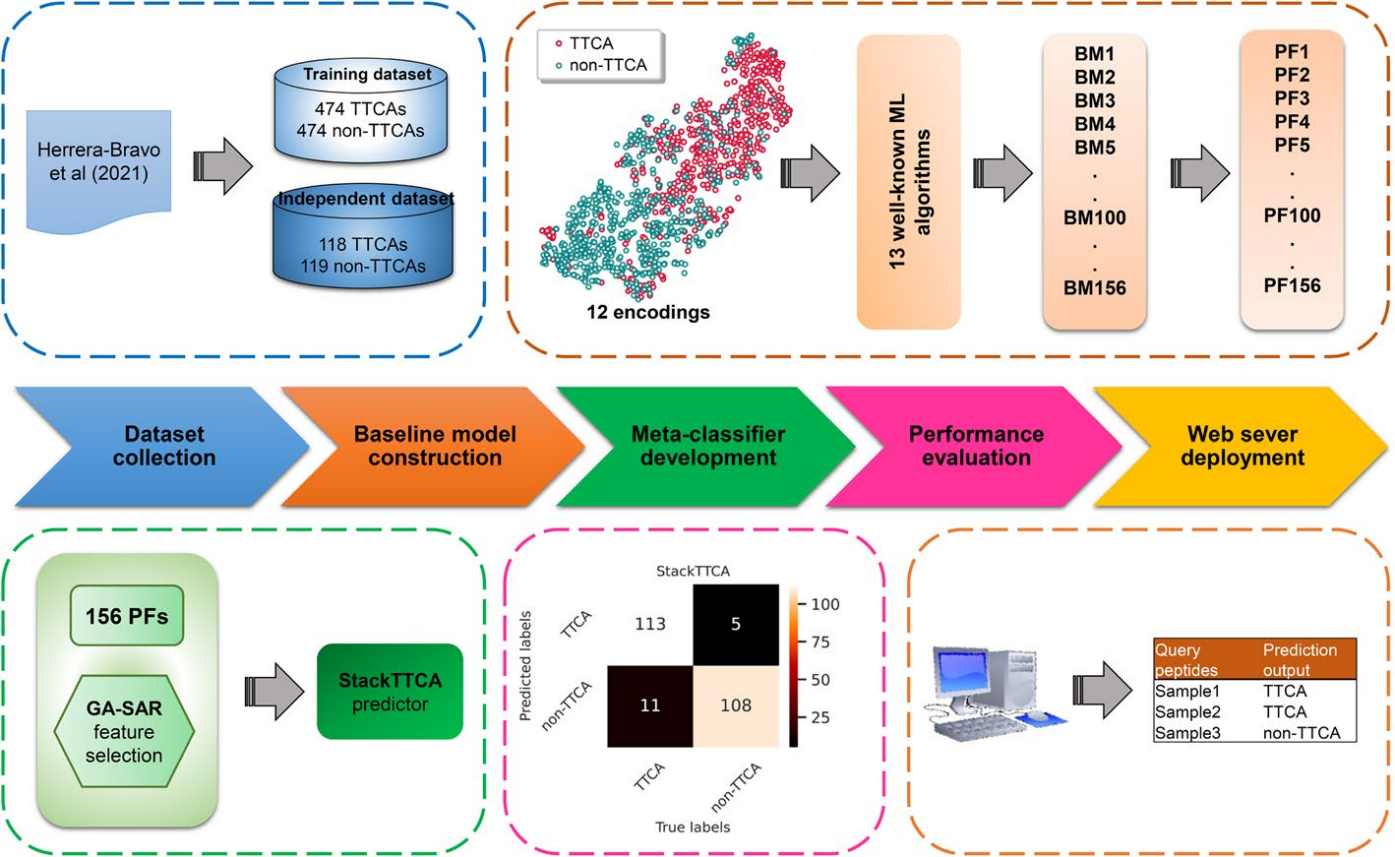
The overall workflow of our proposed approach StackTTCA, which includes five major steps: (i) datasets collection, (ii) baseline model construction, (iii) meta-classifier development, (iv) performance evaluation, and (v) web server deployment

The objective of this study is to present a stacking ensemble learning-based framework, called as StackTTCA, for the precise and comprehensive detection of TTCAs. The procedure of the StackTTCA development is described in Fig. 1. First, we employed 12 different feature encoding schemes from various aspects to extract the information of TTCAs, including composition information, reduced amino acid sequences information, pseudo amino acid composition information, and physicochemical properties. Second, we trained 13 individual ML methods by using each feature encoding. As a result, 156 baseline models were obtained and used to create a 156-D probabilistic feature vector. Finally, the feature selection strategy was utilized to optimize this probabilistic feature vector and then used as the optimal feature vector for the construction of the stacked model. After conducting extensive comparative analysis through an independent test, it was found that StackTTCA demonstrated superior performance in identifying TTCAs when compared to several ML classifiers and existing methods. In order to better understand the remarkable performance of StackTTCA, we have utilized the Shapley Additive exPlanation algorithm to enhance model interpretation and identify the most significant features of StackTTCA. Finally, an online web server (http://2pmlab.camt.cmu.ac.th/StackTTCA) was created to facilitate high-throughput screening of novel TTCAs, maximizing user convenience.

## Materials and methods

### Overall framework of StackTTCA

A comprehensive illustration of the steps involved in the development and performance evaluation of StackTTCA is provided in Fig. 1. Firstly, robust training and independent test datasets were gathered. Subsequently, a collection of baseline models was established by employing 13 machine learning methods in combination with 12 feature encoding techniques. The resulting baseline models were then used to generate a feature vector comprising 156 probabilistic features with a range of 0 to 1. The feature vector was further optimized through a feature selection scheme to construct the meta-classifier, i.e., StackTTCA. The efficacy of the StackTTCA was evaluated using tenfold cross-validation, independent testing, and case studies. Finally, an online web server for StackTTCA was developed to enhance its accessibility and usability.

### Benchmark dataset

In fact, there are two popular benchmark datasets, which were originally collected by Charoenkwan et al. [18] and Herrera-Bravo et al. [19]. However, the dataset in [18] involved incorrect negative samples [19, 22]. Thus, in this study, we employed the remaining benchmark dataset for assessing the performance of our proposed approach. This dataset was used for training several existing methods (i.e., iTTCA-Hybrid [18], TAP1.0 [19], iTTCA-RF [20], and PSRTTCA [22]). To be specific, the number of unique TTCAs and unique non-TTCAs are 592 and 593, which are considered as positive and negative samples, respectively. The training dataset from Herrera-Bravo et al. [19] were constructed by randomly selecting 474 TTCAs and 474 non-TTCAs, while the remaining TTCAs and non-TTCAs were employed as the independent test dataset.

### Stacking ensemble learning-based framework

Instead of simply selecting an optimal single ML model, this study aims to build a stacking ensemble learning-based framework [14–16, 23, 24] by taking advantage of several ML models for the improved prediction performance of TTCAs. Figure 1 illustrates the overall framework of StackTTCA. It comprises of three main steps, including baseline model construction, probabilistic feature optimization, and meta-classifier development. In brief, we first applied the state-of-the-art ML methods and feature encoding schemes to create a pool of baseline models. Second, the output of these baseline models are generated and optimized using the feature selection scheme. Finally, the optimal feature set is employed to develop a meta-classifier.

At the first step, TTCAs and non-TTCAs were encoded based on 12 different feature encoding schemes (see Table 2). After that, we trained 13 individual ML methods by using each feature encoding scheme. As a result, we obtained 156 baseline models (13 ML × 12 encoding). In addition, we employed a grid search to determine the optimal parameters of ADA, ET, LGBM, LR, MLP, RF, SVMLN, SVMRBF, and XGB classifiers in conjunction with the tenfold cross-validation procedure to maximize their performances (see Additional file 1: Table S1). All the baseline models were created using the Scikit-learn v0.24.1 package [25].

**Table 2:** Summary of 12 different feature encodings along with their corresponding description and dimension

Order	Descriptors^a^	Description	Dimension	References
1	AAC	Frequency of 20 amino acids	20	[26]
2	AAI	Different biochemical and biophysical properties extracted from the AAindex database	531	[27, 28]
3	APAAC	Amphiphilic pseudo-amino acid composition	22	[27, 28]
4	CTD	Composition, transition and distribution	147	[27, 28]
5	DPC	Frequency of 400 dipeptides	400	[27, 28]
6	PCP	Different biochemical and biophysical properties extracted from the AAindex database	11	[27, 28]
7	PAAC	Pseudo amino acid composition	21	[27, 28]
8	RSacid	Reduced amino acid sequences according to acidity	32	[29]
9	RScharge	Reduced amino acid sequences according to charge	50	[29]
10	RSDHP	Reduced amino acid sequences according to DHP	32	[29]
11	RSpolar	Reduced amino acid sequences according to polarity	32	[29]
12	RSsecond	Reduced amino acid sequences according to secondary structure	40	[29]

^a^*AAC* Amino acid composition, *AAI* Amino acid index database, *APAAC* Pseudo amino acid composition, *CTD* Composition–transition–distribution, *DPC* Dipeptide composition, *PCP* Physicochemical properties, *PACC* Pseudo amino acid composition, *RS* Reduced amino acid sequences

At the second step, we conducted the tenfold cross-validation procedure for each baseline model to generate a new probabilistic feature for extracting the crucial information of TTCAs. After performing this process, we obtained a 156-D probabilistic feature vector (APF). The APF can be represented by

1 $${\text{APF}} = \left\{ {{\text{PF}}_{1,1} , {\text{PF}}_{1,2} ,{\text{PF}}_{1,3} , \ldots ,{\text{PF}}_{{{\text{i}},{\text{j}}}} , \ldots ,{\text{PF}}_{13,12} } \right\}$$

where ${\text{PF}}_{{{\text{i}},{\text{j}}}}$ is the probabilistic feature (PF) generated from the *i*th ML method in conjunction with the *j*th feature encoding*.* Although the dimension of the APF is 156, some of them are not effective and provide noisy information. Therefore, we conducted the feature optimization process based on our developed genetic algorithm (GA), termed (GA-SAR) [27, 30–32], for determining *m* import PFs (*m* < 156). The *m*-D probabilistic feature vector is referred as BPF. The chromosomes of GA-SAR consist of two parts, including binary and parametric genes. Herein, the parameters and their values for the GA-SAR contain *r*_*begin*_ = 5, *m*_*stop*_ = 20, *P*_*m*_ = 0.05, and *Pop* = 20 [27, 28, 32]. The procedure of the feature importance selection based on the GA-SAR method is described as follows. First, we randomly constructed a population of *Pop* individuals and comprehensively evaluated the performance of all *Pop* individuals using the fitness function and the tenfold cross-validation scheme. Second, we used the tournament selection to obtain the best *Pop* for the construction of a mating pool. Third, we performed the self-assessment-report operation (SAR) between the best *Pop* and each other individual *Pop* to obtain the new children. In this study, we treated 20 generations as the stop condition. Further information regarding this algorithm has been provided in our previous studies [32–35].

In the last step, we used ET method as the meta-classifier (called mET) for the development of the stacked model. We trained individually stacking ensemble models by using two probabilistic feature vector, including APF and BPF. The binary and parametric genes of the mET predictor consisted of *n* = 156 PFs and n_estimators $\in$ {20, 50, 100, 200, 500} (see Additional file 1: Table S1). Here, we selected the best-performing feature vector in terms of MCC in order to construct StackTTCA.

### Evaluation metrics

In order to show the effectiveness of our proposed approach, its prediction performance was assessed by using four standard evaluation metrics, including ACC, MCC, specificity (Sp) and sensitivity (Sn) [36]. These evaluation metrics are computed as follows:

2 $${\text{ACC}} = \frac{{{\text{TP}} + {\text{TN}}}}{{\left( {{\text{TP}} + {\text{TN}} + {\text{FP}} + {\text{FN}}} \right)}}$$

3 $${\text{MCC}} = \frac{{{\text{TP}} \times {\text{TN}} - {\text{FP}} \times {\text{FN}}}}{{\sqrt[{}]{{\left( {{\text{TP}} + {\text{FP}}} \right)\left( {{\text{TP}} + {\text{FN}}} \right)\left( {{\text{TN}} + {\text{FP}}} \right)\left( {{\text{TN}} + {\text{FN}}} \right)}}}}$$

4 $${\text{Sp}} = \frac{{{\text{TN}}}}{{\left( {{\text{TN}} + {\text{FP}}} \right)}}$$

5 $${\text{Sn}} = \frac{{{\text{TP}}}}{{\left( {{\text{TP}} + {\text{FN}}} \right)}}$$

where TN and TP are the number of negative and positive samples predicted to be negative and positive, respectively. In the meanwhile, FN and FP are the number of positive and negative samples predicted to be negative and positive, respectively [37–40]. Furthermore, we utilized area under the receiver operating characteristics (ROC) curve (AUC) to assess the robustness of the model [41, 42].

## Results

### Optimization of stacked models

In our stacking framework, two new probabilistic feature vectors (i.e., APF and BPF) were generated based on a pool of ML classifiers and then used to construct stacked models. Here, we assessed and compared the impact of these two vectors using mET classifiers in TTCA identification. As mentioned above, the APF was represented by the 156-D probabilistic feature vector, while the BPF was obtained by using the GA-SAR method for the selection of *m* import PFs. After performing the feature selection, the optimal number of *m* was 10. Specifically, the 10 import PFs were generated based on 10 different ML classifiers, including ET-RSAcid, LR-RSAcid, ET-DPC, SVMLN-CTD, XGB-CTD, ET-APAAC, ADA-APAAC, RF-PCP, SVMLN-AAI, and PLS-AAI. The performance comparison results between the APF and BPF are shown in Table 3. In case of the tenfold cross-validation results, it could be noticed that both APF and BPF exhibits impressive overall performance in terms of ACC, MCC, and AUC with ranges of 0.867–0.879, 0.737–0.760, and 0.933–0.935, respectively. In the meanwhile, we observed that the BPF outperformed the APF in terms of all five metrics used. Furthermore, on the independent test dataset, the BPF’s ACC, MCC, and Sn were 3.38, 6.85, and 5.08%, respectively, higher than the APF. As a results, the BPF was selected to construct our final stacked model.

**Table 3:** Cross-validation and independent test results for ET classifiers trained with three different features

Evaluation strategy	Feature	Number of feature	ACC	Sn	Sp	MCC	AUC
Cross-validation	APF	156	0.867	0.884	0.850	0.737	0.933
	BPF	10	0.879	0.896	0.861	0.760	0.935
Independent test	APF	156	0.899	0.907	0.891	0.798	0.958
	BPF	10	0.932	0.958	0.908	0.866	0.962

### Performance comparison with other ensemble strategies

To verify the necessity of the stacking strategy, we compared its performance with that of related ensemble strategies [16, 33, 34, 43], namely, the average scoring and majority voting. In brief, the average scoring and majority voting involves using the prediction outputs from 156 baseline models to create corresponding ensemble models by averaging and voting the probabilistic scores, respectively. Table 4 summarizes the performance comparison of different models trained based on various ensemble strategies. In Table 4, both the cross-validation and independent test results demonstrate that the stacking strategy exhibits impressive overall performance across all five evaluation metrics. For example, in terms of the independent test results, the stacking strategy outperforms the two compared ensemble strategies by 10.92–11.77, 19.53–20.38, and 21.47–23.42% in ACC, Sn, and MCC, respectively. These results indicate that the stacking strategy is an effective approach for improving the prediction of TTCAs.

**Table 4:** Performance comparison of different models trained based on different ensemble strategies

Evaluation strategy	Ensemble strategy	ACC	Sn	Sp	MCC	AUC
Cross-validation	Average score	0.792	0.726	0.858	0.589	0.887
	Majority voting	0.782	0.734	0.831	0.568	0.887
	Stacking	0.879	0.896	0.861	0.760	0.935
Independent test	Average score	0.823	0.754	0.891	0.651	0.910
	Majority voting	0.814	0.763	0.866	0.632	0.905
	Stacking	0.932	0.958	0.908	0.866	0.962

### Performance comparison with conventional ML classifiers

**Fig. 2:**
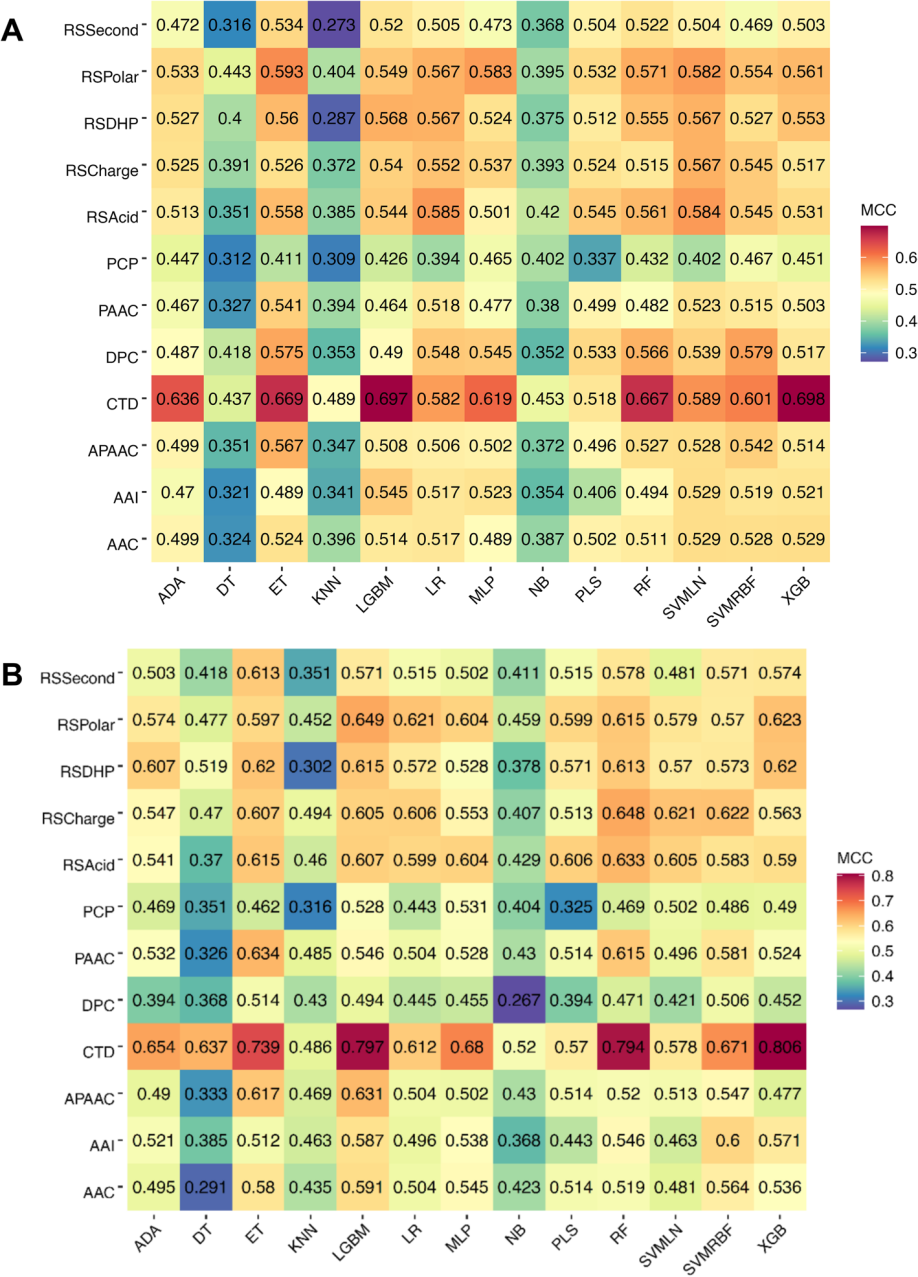
MCC values of 156 baseline models in terms of tenfold cross-validation (**A**) and independent (**B**) tests

**Fig. 3:**
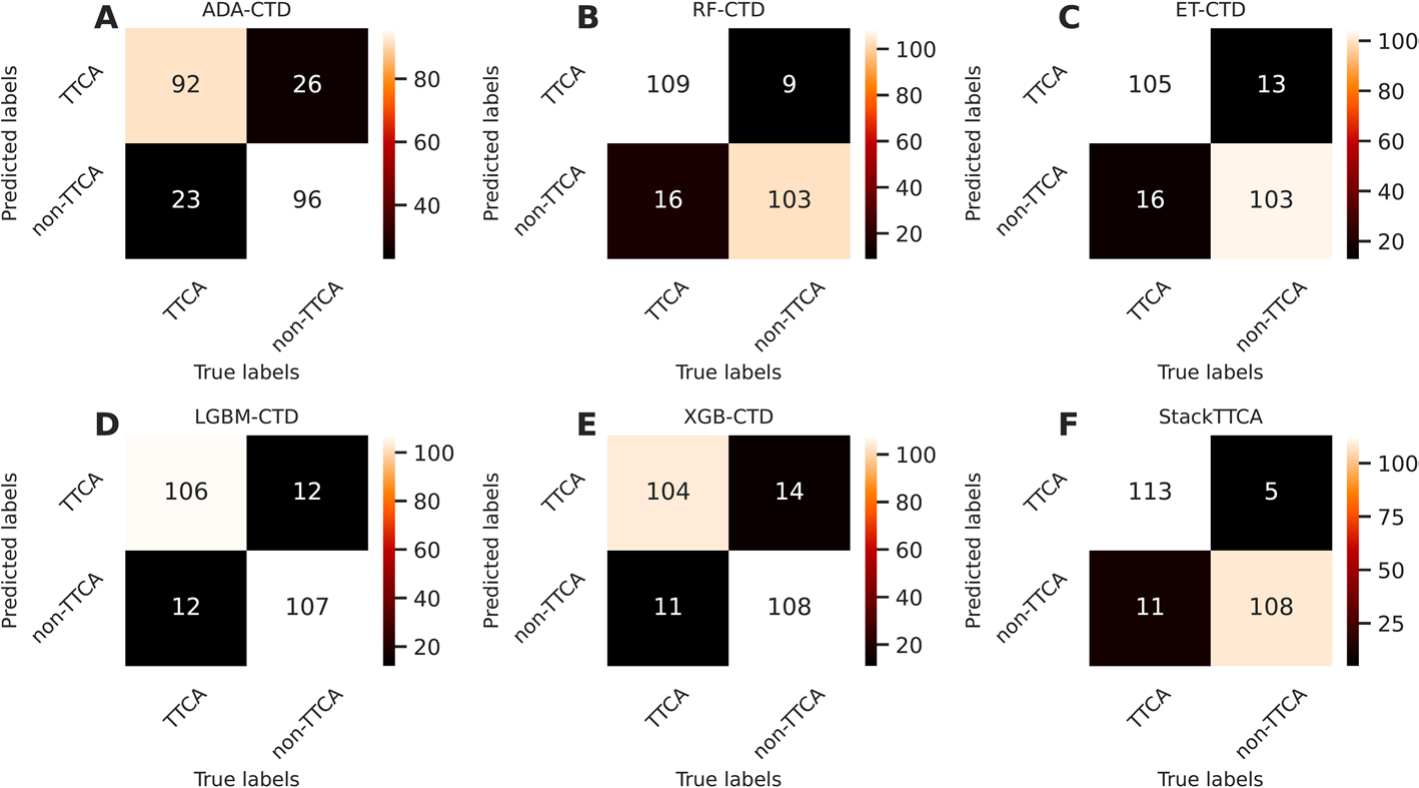
Confusion matrices of StackTTCA and top five ML classifiers in terms of the independent test dataset. ADA-CTD (**A**), RF-CTD (**B**), ET-CTD (**C**), LGBM-CTD (**D**), XGB-CTD (**E**), StackTTCA (**F**)

**Fig. 4:**
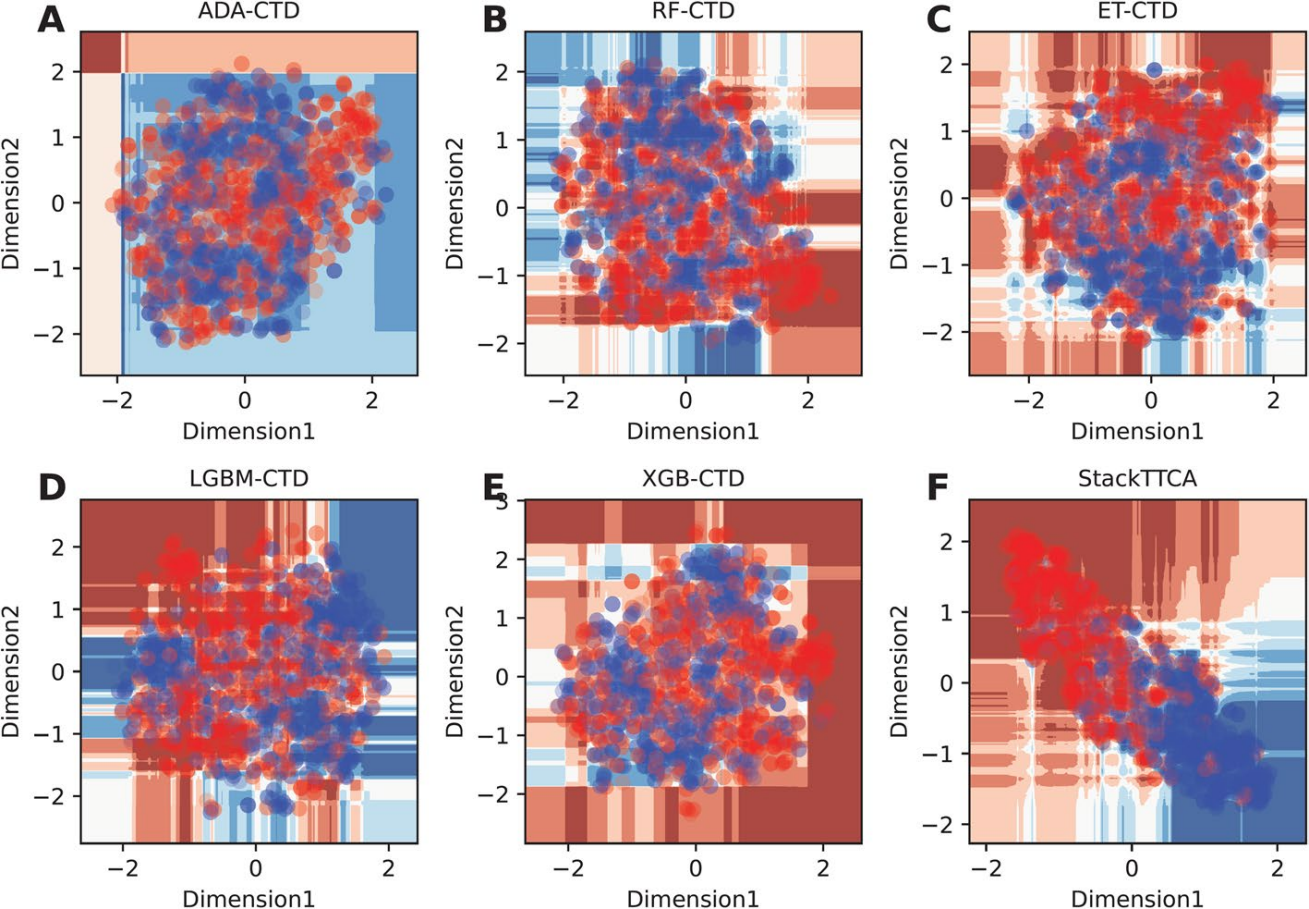
t-distributed stochastic neighbor embedding (t-SNE) distribution of positive and negative samples on the training dataset, where TTCAs and non-TTCAs are represented with red and blue dots, respectively. ADA-CTD (**A**), RF-CTD (**B**), ET-CTD (**C**), LGBM-CTD (**D**), XGB-CTD (**E**), StackTTCA (**F**)

In this section, the performance of all 156 constituent baseline models are assessed and presented in Fig. 2 and Additional file 1: Tables S2 and S3. As shown in Fig. 2, we noticed that the top ten baseline models having the highest MCC consist of XGB-CTD, LGBM-CTD, ET-CTD, RF-CTD, ADA-CTD, MLP-CTD, SVMRBF-CTD, ET-RSPolar, SVMLN-CTD, and LR-RSAcid. In the meanwhile, eight out of the top ten baseline models were developed based on the CTD descriptor, highlighting that the CTD descriptor was crucial in TTCA identification. For the XGB-CTD’s cross-validation results, Table 5 shows that this classifier exhibits the highest ACC and MCC of 0.848 and 0.698, respectively. XGB-CTD still outperformed other compared ML classifiers in terms of ACC and MCC on the independent test dataset. This evidence implies that XGB-CTD was the best ML classifier among all the compared ML classifiers. Therefore, we further compared the performance of StackTTCA against the top five baseline models (i.e., XGB-CTD, LGBM-CTD, ET-CTD, RF-CTD, and ADA-CTD) to elucidate the advantages of the stacking strategy (Table 4). StackTTCA demonstrated superior performance on both the training and independent test datasets, outperforming all other methods across all five evaluation metrics. Impressively, in the context of the independent test dataset, the ACC, Sn, and MCC of StackTTCA were 2.95, 4.24, and 5.99%, respectively, higher than XGB-CTD. Additionally, among the top five baseline models, StackTTCA exhibited the highest number of true positives and the lowest number of false negatives (Fig. 3). Furthermore, to understand the reason behind the better performance of StackTTCA, we utilized t-SNE to generate six boundary plots for our model and the top five baseline models [44, 45]. These plots depict TTCAs and non-TTCAs as red and blue dots, respectively. The visualization in Fig. 4 reveals that StackTTCA accurately classified majority of dots, whereas several dots from the top five baseline models were misclassified. Taking into account both cross-validation and independent test outcomes, StackTTCA exhibits improved and consistent prediction performance compared to several conventional ML classifiers.

**Table 5:** Performance comparison of StackTTCA and top five ML classifiers

Evaluation strategy	Method	ACC	Sn	Sp	MCC	AUC
Cross-validation	ADA-CTD	0.816	0.829	0.803	0.636	0.893
	RF-CTD	0.832	0.854	0.810	0.667	0.912
	ET-CTD	0.833	0.848	0.818	0.669	0.917
	LGBM-CTD	0.847	0.861	0.833	0.697	0.921
	XGB-CTD	0.848	0.852	0.843	0.698	0.920
	StackTTCA	0.879	0.896	0.861	0.760	0.935
Independent test	ADA-CTD	0.827	0.822	0.832	0.654	0.918
	RF-CTD	0.895	0.949	0.840	0.794	0.942
	ET-CTD	0.869	0.881	0.857	0.739	0.945
	LGBM-CTD	0.899	0.898	0.899	0.797	0.951
	XGB-CTD	0.903	0.915	0.891	0.806	0.946
	StackTTCA	0.932	0.958	0.908	0.866	0.962

### Performance comparison with state-of-the-art methods

**Fig. 5:**
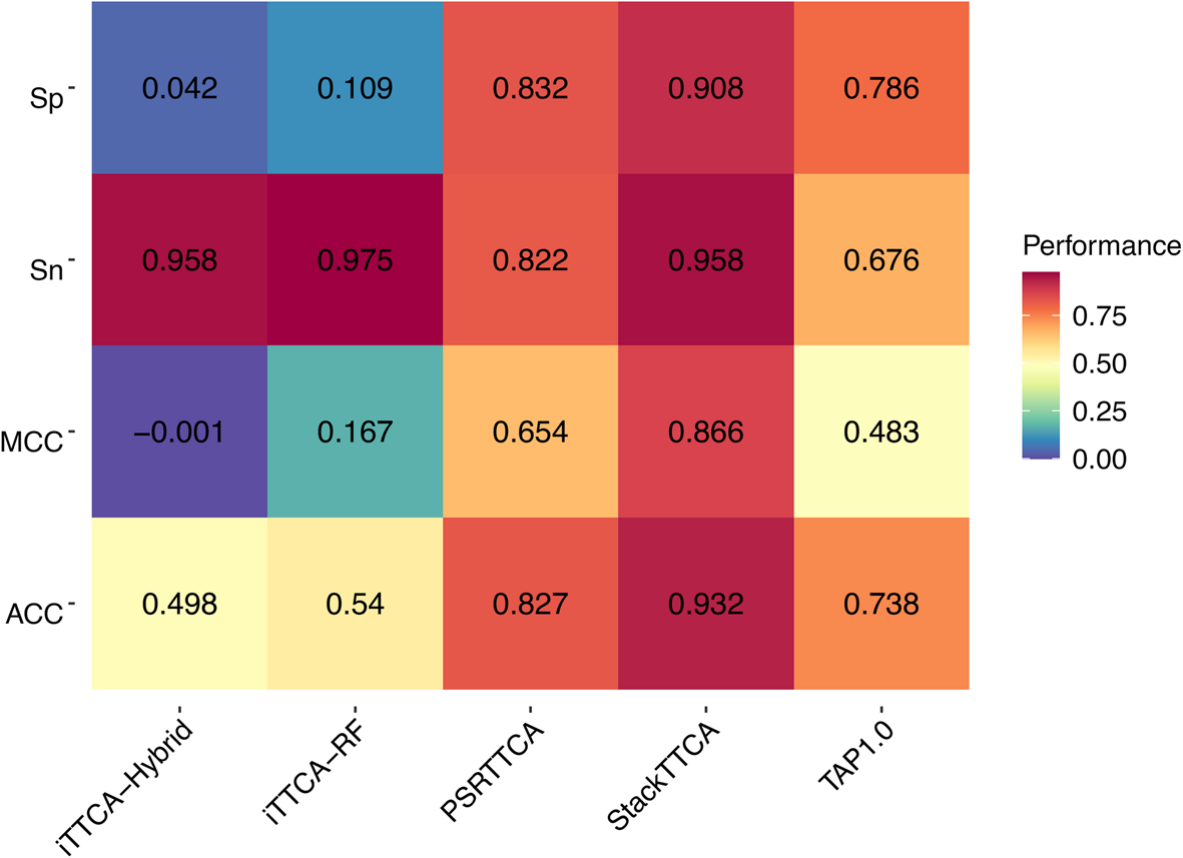
Heat-map of the prediction performance of StackTTCA and the state-of-the-art methods in terms of the independent test dataset

In this section, we compared the performance of StackTTCA against the state-of-the-art methods by conducting an independent test. To conduct a fair performance comparison, the state-of-the-art methods involving iTTCA-Hybrid [18], TAP1.0 [19], iTTCA-RF [20], and PSRTTCA [22] were selected for the comparative analysis herein. All prediction performances of these four methods are directly obtained from the PSRTTCA study [22]. Figure 5 and Table 6 show the performance comparison results of StackTTCA and the four state-of-the-art methods. Among these four compared methods, the most effective one was PSRTTCA, which clearly outperformed other related methods. By comparing with PSRTTCA, StackTTCA achieved a better performance in terms of ACC, Sn, Sp, and MCC. To be specific, the ACC, Sn, Sp, and MCC of StackTTCA was 10.55, 13.56, 7.56, and 21.21%, respectively, higher than PSRTTCA. In addition, we also performed case studies to verify the predictive reliability in realistic scenarios. All 73 experimentally verified TTCAs were retrieved from the PSRTTCA study [22]. Additional file 1: Table S4 lists the prediction results of StackTTCA and the four compared methods. As can be seen from Additional file 1: Table S4, StackTTCA secured the best performance in terms of the case studies. Specifically, 60 out of 73 TTCAs (ACC of 0.822) were correctly predicted by StackTTCA, while the four compared methods could correctly predict 45 – 55 peptide sequences to be TTCAs (ACC of 0.616–0.753). These results highlight the effectiveness and generalization ability of the proposed model, highlighting that StackTTCA can help to precisely and rapidly identify true TTCAs for follow-up experimental verification.

**Table 6:** Performance comparison of StackTTCA and the state-of-the-art methods on the independent test dataset

Method	#Feature	ACC	Sn	SP	MCC
iTTCA-Hybrid	224	0.498	0.958	0.042	− 0.001
TAP1.0	10	0.738	0.676	0.786	0.483
iTTCA-RF	341	0.540	0.975	0.109	0.167
PSRTTCA	7	0.827	0.822	0.832	0.654
StackTTCA	10	0.932	0.958	0.908	0.866

### Feature importance analysis

**Fig. 6:**
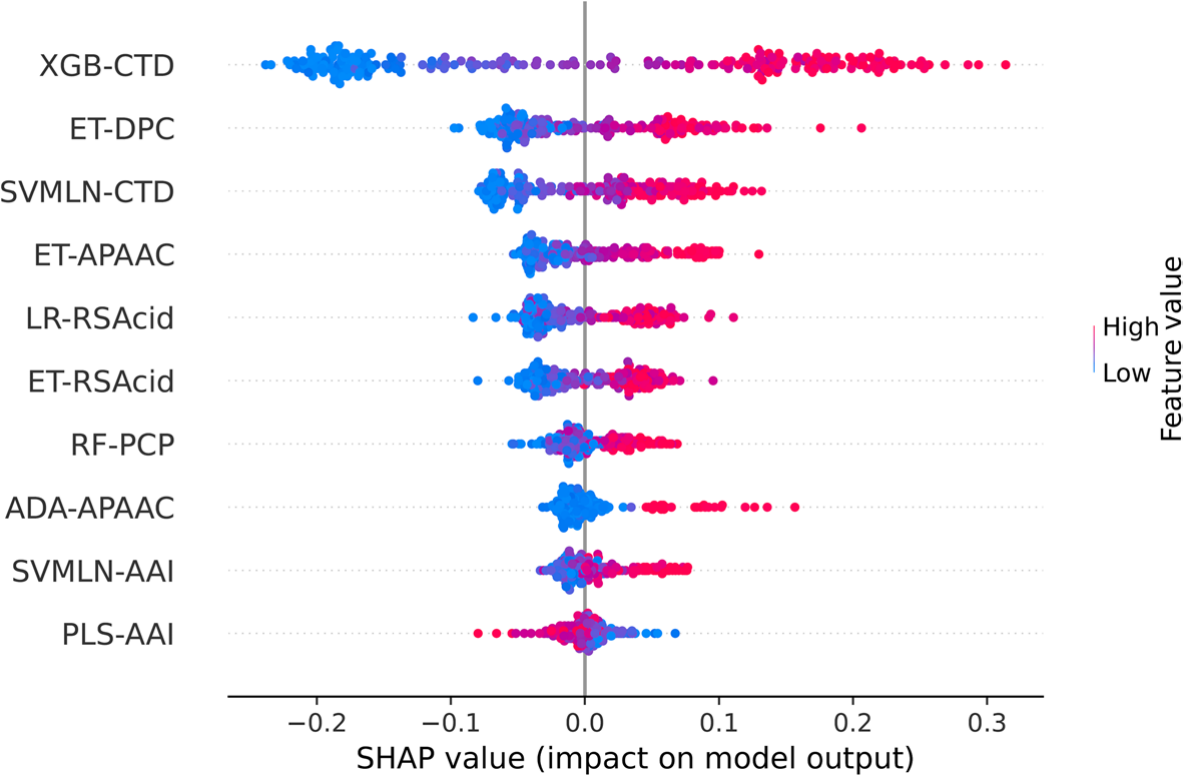
Feature importance from StackTTCA, where positive and negative SHAP values indicate the high probability that the prediction outputs are TTCA and non-TTCA, respectively

In this section, we explore the impact of the 10 essential PFs used to create StackTTCA. We used the SHAP method to interpret the StackTTCA’s TTCAs identification. These PFs were generated from 10 different ML classifiers that were selected using the GA-SAR method. The classifiers used were ET-RSAcid, LR-RSAcid, ET-DPC, SVMLN-CTD, XGB-CTD, ET-APAAC, ADA-APAAC, RF-PCP, SVMLN-AAI, and PLS-AAI. Figure 6 illustrates the feature ranking of the 10 essential PFs based on their Shapley values. A positive SHAP value indicates a high likelihood of the prediction outputs being TTCA, while a negative value suggests a low probability of the outputs being TTCA. The top five crucial PFs were determined to be those based on XGB-CTD, ET-DPC, SVMLN-CTD, ET-APAAC, and LR-RSAcid, all of which exhibited positive SHAP values. Consequently, XGB-CTD had a relatively high probabilistic score for most TTCAs, while it had a relatively low score for most non-TTCAs. In contrast, PLS-AAI had a relatively high score for most non-TTCAs and a relatively low score for most TTCAs.

## Discussion

Discovery and characterization of new TTCAs via experimental technologies are expensive and time-consuming. Therefore, computational approaches that can identify TTCAs using sequence information alone are highly desirable to facilitate community-wide efforts in analyzing and characterizing TTCAs. Although a variety of computational approaches have been proposed for TTCA identification, their performance is still not satisfactory. To overcome this shortcoming, this study presents StackTTCA, a stacking ensemble learning-based framework, for accurately identifying TTCAs and facilitating their large-scale characterization. In the present study, we conducted the three comparative experiments to compare the performance of StackTTCA against conventional ML classifiers, related ensemble strategies, and existing state-of-the-art methods. These experiments aimed to reveal the effectiveness and robustness of our proposed approach. The comparative experiments on the independent test dataset and case studies indicate that StackTTCA is capable of providing more accurate and stable prediction performance. Although the developed StackTTCA approach achieves improvement in TTCA identification, this study still has some shortcomings that can be addressed in future work. Firstly, the limited number of available TTCAs might restrict the prediction performance [46, 47]. Thus, we are motivated to collect additional TTCAs and combine them to construct an up-to-date dataset. Secondly, the discriminative power of the feature representation directly influences the model’s performance. In the future, we plan to combine our probabilistic features with other informative and powerful features, such as fastText, GloVe, and Word2Vec [48, 49].

## Conclusion

In this research, we have introduced a novel stacking ensemble learning framework, called StackTTCA, for identifying TTCAs accurately and facilitating the large-scale characterization. The major contributions of StackTTCA are as follows: (i) StackTTCA utilized various feature encoding methods from different perspectives to extract information related to TTCAs, including composition information, reduced amino acid sequence information, pseudo amino acid composition information, and physicochemical properties. Thirteen individual ML methods were used to establish 156 different baseline models, which generated a 156-D probabilistic feature vector. This feature vector was optimized and used to construct the optimal stacked model; (ii) Through a series of benchmarking experiments, we demonstrated that StackTTCA outperformed several conventional ML classifiers and existing methods in terms of independent testing, achieving an accuracy of 0.932 and Matthew's correlation coefficient of 0.866; (iii) We employed the interpretable SHAP method to analyze and elucidate the identification of TTCAs by StackTTCA; and (iv) To facilitate high-throughput screening of new TTCAs, we developed an online web server (http://2pmlab.camt.cmu.ac.th/StackTTCA) for user convenience.

## Supplementary Information

**Additional file 1: Table S1.** Hyperparameter search details used for the construction of nine ML-based classifiers. **Table S2.** Cross-validation results of 156 baseline models as developed with 13 ML algorithms and 12 feature encoding schemes. **Table S3.** Independent test results of 156 baseline models as developed with 13 ML algorithms and 12 feature encoding schemes. **Table S4.** Detailed prediction results of TAP 1.0, iTTCA-Hybrid, iTTCA-RF, PSATTCA, and StackTTCA on case studies.

## Data Availability

All the data used in this study are available at http://2pmlab.camt.cmu.ac.th/StackTTCA.

## Abbreviations

TAAs: Tumor associated antigens
TAs: Tumor antigens
TSAs: Tumor specific antigens
DCs: Dendritic cells
MHC-I: Histocompatibility complex class I
IEDB: Immune epitope database
TTCAs: Tumor T-cell antigens
ML: Machine learning
ET: Extremely randomized trees
QDA: Quadratic discriminant analysis
RF: Random forest
SVM: Support vector machine
SCM: Scoring card method
MCC: Matthew’s correlation coefficient
ACC: Accuracy
ADA: AdaBoost
DT: Decision tree
KNN: K-nearest neighbor
LGBM: Light gradient boosting machine
LR: Logistic regression
MLP: Multilayer perceptron
NB: Naive Bayes
PLS: Partial least squares
SVMRBF: Support vector machine with radial basis function
SVMLN: Support vector machine with linear kernels
XGB: Extreme gradient boosting.
PF: Probabilistic feature
GA: Genetic algorithm
SAR: Self-assessment-report operation
Sp: Specificity
Sn: Sensitivity
TP: True positive
FP: False positive
TN: True negative
FN: False negative
ROC: Receiver operating characteristic
AUC: Area under the ROC curve

## References

[1] Ilyas S, Yang JC (2015). Landscape of tumor antigens in T cell immunotherapy. J Immunol.

[2] Zamora AE, Crawford JC, Thomas PG (2018). Hitting the target: how T cells detect and eliminate tumors. J Immunol.

[3] Zhang L, Huang Y, Lindstrom AR, Lin T-Y, Lam KS, Li Y (2019). Peptide-based materials for cancer immunotherapy. Theranostics.

[4] Vermaelen K (2019). Vaccine strategies to improve anti-cancer cellular immune responses. Front Immunol.

[5] Alspach E (2019). MHC-II neoantigens shape tumour immunity and response to immunotherapy. Nature.

[6] Breckpot K, Escors D (2009). Dendritic cells for active anti-cancer immunotherapy: targeting activation pathways through genetic modification. Endocr Metab Immune Disord Drug Targets (Former Curr Drug Targets Immune Endocr Metab Disord).

[7] Miliotou AN, Papadopoulou LC (2018). CAR T-cell therapy: a new era in cancer immunotherapy. Curr Pharm Biotechnol.

[8] Calis JJ (2013). Properties of MHC class I presented peptides that enhance immunogenicity. PLoS Comput Biol.

[9] Chowell D (2015). TCR contact residue hydrophobicity is a hallmark of immunogenic CD8+ T cell epitopes. Proc Natl Acad Sci.

[10] Nishimura Y, Tomita Y, Yuno A, Yoshitake Y, Shinohara M (2015). Cancer immunotherapy using novel tumor-associated antigenic peptides identified by genome-wide cDNA microarray analyses. Cancer Sci.

[11] Vita R (2019). The immune epitope database (IEDB): 2018 update. Nucleic Acids Res.

[12] Olsen LR, Tongchusak S, Lin H, Reinherz EL, Brusic V, Zhang GL (2017). TANTIGEN: a comprehensive database of tumor T cell antigens. Cancer Immunol Immunother.

[13] Zhang G, Chitkushev L, Olsen LR, Keskin DB, Brusic V (2021). TANTIGEN 2.0: a knowledge base of tumor T cell antigens and epitopes. BMC Bioinform.

[14] Wei L, Zhou C, Chen H, Song J, Su R (2018). ACPred-FL: a sequence-based predictor using effective feature representation to improve the prediction of anti-cancer peptides. Bioinformatics.

[15] Rao B, Zhou C, Zhang G, Su R, Wei L (2020). ACPred-Fuse: fusing multi-view information improves the prediction of anticancer peptides. Brief Bioinform.

[16] Qiang X, Zhou C, Ye X, Du P-F, Su R, Wei L (2020). CPPred-FL: a sequence-based predictor for large-scale identification of cell-penetrating peptides by feature representation learning. Brief Bioinform.

[17] Lissabet JFB, Belén LH, Farias JG (2019). TTAgP 1.0: a computational tool for the specific prediction of tumor T cell antigens. Comput Biol Chem.

[18] Charoenkwan P, Nantasenamat C, Hasan MM, Shoombuatong W (2020). iTTCA-Hybrid: improved and robust identification of tumor T cell antigens by utilizing hybrid feature representation. Anal Biochem.

[19] Herrera-Bravo J, Belén LH, Farias JG, Beltrلn JF (2021). TAP 1.0: a robust immunoinformatic tool for the prediction of tumor T-cell antigens based on AAindex properties. Comput Biol Chem.

[20] Jiao S, Zou Q, Guo H, Shi L (2021). iTTCA-RF: a random forest predictor for tumor T cell antigens. J Transl Med.

[21] Zou H, Yang F, Yin Z (2022). iTTCA-MFF: identifying tumor T cell antigens based on multiple feature fusion. Immunogenetics.

[22] Charoenkwan P, Pipattanaboon C, Nantasenamat C, Hasan MM, Moni MA, Shoombuatong W (2023). PSRTTCA: a new approach for improving the prediction and characterization of tumor T cell antigens using propensity score representation learning. Comput Biol Med.

[23] Zhang T, Jia Y, Li H, Xu D, Zhou J, Wang G (2022). CRISPRCasStack: a stacking strategy-based ensemble learning framework for accurate identification of Cas proteins. Brief Bioinform.

[24] Wu H (2022). scHiCStackL: a stacking ensemble learning-based method for single-cell Hi-C classification using cell embedding. Brief Bioinform.

[25] Pedregosa F (2011). Scikit-learn: machine learning in Python. J Mach Learn Res.

[26] Ahmad S (2022). SCORPION is a stacking-based ensemble learning framework for accurate prediction of phage virion proteins. Sci Rep.

[27] Charoenkwan P, Schaduangrat N, Moni MA, Manavalan B, Shoombuatong W (2022). SAPPHIRE: a stacking-based ensemble learning framework for accurate prediction of thermophilic proteins. Comput Biol Med.

[28] Charoenkwan P, Schaduangrat N, Moni MA, Manavalan B, Shoombuatong W (2022). NEPTUNE: a novel computational approach for accurate and large-scale identification of tumor homing peptides. Comput Biol Med.

[29] Xu C, Ge L, Zhang Y, Dehmer M, Gutman I (2017). Computational prediction of therapeutic peptides based on graph index. J Biomed Inform.

[30] Charoenkwan P (2022). AMYPred-FRL is a novel approach for accurate prediction of amyloid proteins by using feature representation learning. Sci Rep.

[31] Charoenkwan P, Schaduangrat N, Moni MA, Shoombuatong W, Manavalan B (2022). Computational prediction and interpretation of druggable proteins using a stacked ensemble-learning framework. Iscience.

[32] Charoenkwan P, Schaduangrat N, Nantasenamat C, Piacham T, Shoombuatong W (2019). iQSP: a sequence-based tool for the prediction and analysis of quorum sensing peptides using informative physicochemical properties. Int J Mol Sci.

[33] Charoenkwan P, Nantasenamat C, Hasan MM, Moni MA, Manavalan B, Shoombuatong W (2021). UMPred-FRL: a new approach for accurate prediction of umami peptides using feature representation learning. Int J Mol Sci.

[34] Charoenkwan P, Nantasenamat C, Hasan MM, Moni MA, Manavalan B, Shoombuatong W (2022). StackDPPIV: a novel computational approach for accurate prediction of dipeptidyl peptidase IV (DPP-IV) inhibitory peptides. Methods.

[35] Charoenkwan P, Schaduangrat N, Lio P, Moni MA, Manavalan B, Shoombuatong W (2022). NEPTUNE: a novel computational approach for accurate and large-scale identification of tumor homing peptides. Comput Biol Med.

[36] Azadpour M, McKay CM, Smith RL (2014). Estimating confidence intervals for information transfer analysis of confusion matrices. J Acoust Soc Am.

[37] Lai H-Y (2019). iProEP: a computational predictor for predicting promoter. Mol Ther Nucl Acids.

[38] Lv H, Dao F-Y, Guan Z-X, Yang H, Li Y-W, Lin H (2021). Deep-Kcr: accurate detection of lysine crotonylation sites using deep learning method. Brief Bioinform.

[39] Lv H, Zhang Z-M, Li S-H, Tan J-X, Chen W, Lin H (2019). Evaluation of different computational methods on 5-methylcytosine sites identification. Brief Bioinform.

[40] Su Z-D (2018). iLoc-lncRNA: predict the subcellular location of lncRNAs by incorporating octamer composition into general PseKNC. Bioinformatics.

[41] Ullah M, Han K, Hadi F, Xu J, Song J, Yu D-J (2021). PScL-HDeep: image-based prediction of protein subcellular location in human tissue using ensemble learning of handcrafted and deep learned features with two-layer feature selection. Brief Bioinform.

[42] Mandrekar JN (2010). Receiver operating characteristic curve in diagnostic test assessment. J Thorac Oncol.

[43] Xie R (2021). DeepVF: a deep learning-based hybrid framework for identifying virulence factors using the stacking strategy. Brief Bioinform.

[44] Van Der Maaten L (2014). Accelerating t-SNE using tree-based algorithms. J Mach Learn Res.

[45] Van der Maaten L, Hinton G (2008). Visualizing data using t-SNE. J Mach Learn Res.

[46] Su R, Hu J, Zou Q, Manavalan B, Wei L (2020). Empirical comparison and analysis of web-based cell-penetrating peptide prediction tools. Brief Bioinform.

[47] Basith S, Manavalan B, Hwan Shin T, Lee G (2020). Machine intelligence in peptide therapeutics: a next-generation tool for rapid disease screening. Med Res Rev.

[48] Lv H, Dao F-Y, Zulfiqar H, Lin H (2021). DeepIPs: comprehensive assessment and computational identification of phosphorylation sites of SARS-CoV-2 infection using a deep learning-based approach. Brief Bioinform.

[49] Charoenkwan P, Nantasenamat C, Hasan MM, Manavalan B, Shoombuatong W (2021). BERT4Bitter: a bidirectional encoder representations from transformers (BERT)-based model for improving the prediction of bitter peptides. Bioinformatics.

