# Supplementary material for: StackTTCA: a stacking ensemble learning-based framework for accurate and high-throughput identification of tumor T cell antigens
Source: BMC Bioinformatics. 2023 Jul 28;24:301. doi: 10.1186/s12859-023-05421-x (PMC10386778; doi:10.1186/s12859-023-05421-x)
Supplement: Supplementary file 1 — Additional file 1: Table S1. Hyperparameter search details used for the construction of nine ML-based classifiers. Table S2. Cross-validation results of 156 baseline models as developed with 13 ML algorithms and 12 feature encoding schemes. Table S3. Independent test results of 156 baseline models as developed with 13 ML algorithms and 12 feature encoding schemes. Table S4. Detailed prediction results of TAP 1.0, iTTCA-Hybrid, iTTCA-RF, PSATTCA, and StackTTCA on case studies. [file 12859_2023_5421_MOESM1_ESM.docx]

**Table S1.** Hyperparameter search details used for the construction of nine ML-based classifiers.

| **Method^a^** | **Parameters** | **Range of parameters** |
| --- | --- | --- |
| ADA | n_estimators | [20, 50, 100, 200, 500] |
| ET | n_estimators | [20, 50, 100, 200, 500] |
| LGBM | n_estimators | [20, 50, 100, 200, 500] |
| LR | Cos | [0.001, 0.01, 0.1, 1, 10, 100] |
| MLP | hidden_layer_sizes | [50, 100, 300, 500] |
| RF | n_estimators | [20, 50, 100, 200, 500] |
| SVMLN | Cost | [2^0^–2^5^] in log_2_ steps |
| SVMRBF | Cost | [2^-4^–2^4^] in log_2_ steps |
| XGB | n_estimators | [20, 50, 100, 200, 500] |

^a^ ADA: AdaBoost, DT: decision tree, ET: extremely randomized trees, KNN: k-nearest neighbor, LGBM: light gradient boosting machine, LR: logistic regression, MLP: multilayer perceptron, NB: naive Bayes, PLS: partial least squares, RF: random forest, SVMRBF: support vector machine with radial basis function, SVMLN: support vector machine with linear kernels, XGB: extreme gradient boosting.

**Table S2** Cross-validation results of 156 baseline models as developed with 13 ML algorithms and 12 feature encoding schemes.

| **Descriptor** | **Method** | **ACC** | **Sn** | **Sp** | **MCC** | **AUC** | **Parameter used** |
| --- | --- | --- | --- | --- | --- | --- | --- |
| AAC | KNN | 0.697 | 0.700 | 0.693 | 0.396 | 0.697 | NA |
|  | ADA | 0.748 | 0.703 | 0.793 | 0.499 | 0.813 | 50 |
|  | DT | 0.661 | 0.654 | 0.668 | 0.324 | 0.662 | NA |
|  | ET | 0.760 | 0.734 | 0.786 | 0.524 | 0.834 | 50 |
|  | LGBM | 0.755 | 0.715 | 0.795 | 0.514 | 0.833 | 20 |
|  | LR | 0.756 | 0.705 | 0.808 | 0.517 | 0.824 | 10 |
|  | MLP | 0.744 | 0.726 | 0.761 | 0.489 | 0.831 | 20 |
|  | NB | 0.645 | 0.317 | 0.975 | 0.387 | 0.807 | NA |
|  | PLS | 0.748 | 0.675 | 0.820 | 0.502 | 0.819 | NA |
|  | RF | 0.755 | 0.770 | 0.740 | 0.511 | 0.828 | 20 |
|  | SVMLN | 0.761 | 0.709 | 0.814 | 0.529 | 0.822 | 32 |
|  | SVMRBF | 0.761 | 0.717 | 0.805 | 0.528 | 0.833 | 1 |
|  | XGB | 0.763 | 0.707 | 0.818 | 0.529 | 0.839 | 50 |
| AAI | KNN | 0.670 | 0.646 | 0.693 | 0.341 | 0.669 | NA |
|  | ADA | 0.734 | 0.694 | 0.774 | 0.470 | 0.801 | 20 |
|  | DT | 0.660 | 0.686 | 0.634 | 0.321 | 0.661 | NA |
|  | ET | 0.743 | 0.724 | 0.761 | 0.489 | 0.813 | 50 |
|  | LGBM | 0.771 | 0.732 | 0.810 | 0.545 | 0.849 | 100 |
|  | LR | 0.756 | 0.705 | 0.808 | 0.517 | 0.824 | 100 |
|  | MLP | 0.759 | 0.728 | 0.791 | 0.523 | 0.845 | 20 |
|  | NB | 0.674 | 0.591 | 0.757 | 0.354 | 0.722 | NA |
|  | PLS | 0.701 | 0.692 | 0.710 | 0.406 | 0.760 | NA |
|  | RF | 0.746 | 0.728 | 0.763 | 0.494 | 0.826 | 200 |
|  | SVMLN | 0.761 | 0.705 | 0.818 | 0.529 | 0.821 | 16 |
|  | SVMRBF | 0.757 | 0.709 | 0.805 | 0.519 | 0.836 | 2 |
|  | XGB | 0.759 | 0.732 | 0.786 | 0.521 | 0.838 | 200 |
| APAAC | KNN | 0.673 | 0.682 | 0.664 | 0.347 | 0.673 | NA |
|  | ADA | 0.748 | 0.705 | 0.791 | 0.499 | 0.811 | 20 |
|  | DT | 0.674 | 0.659 | 0.689 | 0.351 | 0.674 | NA |
|  | ET | 0.783 | 0.757 | 0.808 | 0.567 | 0.849 | 200 |
|  | LGBM | 0.752 | 0.709 | 0.795 | 0.508 | 0.835 | 20 |
|  | LR | 0.751 | 0.705 | 0.797 | 0.506 | 0.826 | 10 |
|  | MLP | 0.751 | 0.743 | 0.759 | 0.502 | 0.823 | 50 |
|  | NB | 0.640 | 0.312 | 0.968 | 0.372 | 0.809 | NA |
|  | PLS | 0.745 | 0.679 | 0.810 | 0.496 | 0.822 | NA |
|  | RF | 0.762 | 0.734 | 0.791 | 0.527 | 0.830 | 50 |
|  | SVMLN | 0.761 | 0.709 | 0.814 | 0.528 | 0.825 | 2 |
|  | SVMRBF | 0.769 | 0.730 | 0.808 | 0.542 | 0.836 | 1 |
|  | XGB | 0.753 | 0.673 | 0.833 | 0.514 | 0.823 | 20 |
| CTD | KNN | 0.742 | 0.686 | 0.799 | 0.489 | 0.742 | NA |
|  | ADA | 0.816 | 0.829 | 0.803 | 0.636 | 0.893 | 100 |
|  | DT | 0.718 | 0.720 | 0.717 | 0.437 | 0.719 | NA |
|  | ET | 0.833 | 0.848 | 0.818 | 0.669 | 0.917 | 200 |
|  | LGBM | 0.847 | 0.861 | 0.833 | 0.697 | 0.921 | 100 |
|  | LR | 0.790 | 0.795 | 0.784 | 0.582 | 0.879 | 1 |
|  | MLP | 0.807 | 0.791 | 0.822 | 0.619 | 0.885 | 200 |
|  | NB | 0.726 | 0.709 | 0.742 | 0.453 | 0.792 | NA |
|  | PLS | 0.758 | 0.762 | 0.755 | 0.518 | 0.826 | NA |
|  | RF | 0.832 | 0.854 | 0.810 | 0.667 | 0.912 | 200 |
|  | SVMLN | 0.793 | 0.778 | 0.807 | 0.589 | 0.875 | 2 |
|  | SVMRBF | 0.799 | 0.793 | 0.805 | 0.601 | 0.884 | 4 |
|  | XGB | 0.848 | 0.852 | 0.843 | 0.698 | 0.920 | 200 |
| DPC | KNN | 0.675 | 0.736 | 0.613 | 0.353 | 0.675 | NA |
|  | ADA | 0.742 | 0.741 | 0.744 | 0.487 | 0.796 | 100 |
|  | DT | 0.708 | 0.707 | 0.708 | 0.418 | 0.708 | NA |
|  | ET | 0.787 | 0.766 | 0.808 | 0.575 | 0.845 | 50 |
|  | LGBM | 0.744 | 0.781 | 0.706 | 0.490 | 0.814 | 200 |
|  | LR | 0.773 | 0.766 | 0.780 | 0.548 | 0.839 | 0.1 |
|  | MLP | 0.772 | 0.764 | 0.780 | 0.545 | 0.841 | 50 |
|  | NB | 0.626 | 0.283 | 0.971 | 0.352 | 0.628 | NA |
|  | PLS | 0.766 | 0.751 | 0.780 | 0.533 | 0.843 | NA |
|  | RF | 0.782 | 0.775 | 0.788 | 0.566 | 0.849 | 200 |
|  | SVMLN | 0.769 | 0.749 | 0.789 | 0.539 | 0.827 | 2 |
|  | SVMRBF | 0.789 | 0.785 | 0.793 | 0.579 | 0.866 | 2 |
|  | XGB | 0.757 | 0.787 | 0.727 | 0.517 | 0.839 | 200 |
| PAAC | KNN | 0.696 | 0.686 | 0.706 | 0.394 | 0.696 | NA |
|  | ADA | 0.732 | 0.679 | 0.784 | 0.467 | 0.799 | 20 |
|  | DT | 0.663 | 0.658 | 0.668 | 0.327 | 0.663 | NA |
|  | ET | 0.770 | 0.745 | 0.795 | 0.541 | 0.840 | 100 |
|  | LGBM | 0.731 | 0.698 | 0.763 | 0.464 | 0.805 | 20 |
|  | LR | 0.756 | 0.696 | 0.816 | 0.518 | 0.824 | 10 |
|  | MLP | 0.737 | 0.711 | 0.763 | 0.477 | 0.818 | 20 |
|  | NB | 0.643 | 0.316 | 0.970 | 0.380 | 0.810 | NA |
|  | PLS | 0.746 | 0.669 | 0.822 | 0.499 | 0.819 | NA |
|  | RF | 0.740 | 0.717 | 0.763 | 0.482 | 0.819 | 200 |
|  | SVMLN | 0.758 | 0.703 | 0.814 | 0.523 | 0.822 | 32 |
|  | SVMRBF | 0.755 | 0.709 | 0.801 | 0.515 | 0.835 | 1 |
|  | XGB | 0.749 | 0.692 | 0.805 | 0.503 | 0.827 | 50 |
| PCP | KNN | 0.654 | 0.629 | 0.679 | 0.309 | 0.654 | NA |
|  | ADA | 0.722 | 0.690 | 0.755 | 0.447 | 0.793 | 50 |
|  | DT | 0.655 | 0.658 | 0.651 | 0.312 | 0.657 | NA |
|  | ET | 0.705 | 0.667 | 0.742 | 0.411 | 0.759 | 200 |
|  | LGBM | 0.712 | 0.677 | 0.746 | 0.426 | 0.779 | 50 |
|  | LR | 0.696 | 0.675 | 0.717 | 0.394 | 0.756 | 1 |
|  | MLP | 0.730 | 0.679 | 0.780 | 0.465 | 0.804 | 100 |
|  | NB | 0.699 | 0.654 | 0.744 | 0.402 | 0.757 | NA |
|  | PLS | 0.667 | 0.644 | 0.691 | 0.337 | 0.736 | NA |
|  | RF | 0.715 | 0.675 | 0.755 | 0.432 | 0.779 | 50 |
|  | SVMLN | 0.698 | 0.624 | 0.772 | 0.402 | 0.760 | 2 |
|  | SVMRBF | 0.729 | 0.641 | 0.816 | 0.467 | 0.790 | 2 |
|  | XGB | 0.722 | 0.652 | 0.793 | 0.451 | 0.794 | 100 |
| RSAcid | KNN | 0.692 | 0.677 | 0.706 | 0.385 | 0.692 | NA |
|  | ADA | 0.755 | 0.715 | 0.795 | 0.513 | 0.840 | 20 |
|  | DT | 0.675 | 0.681 | 0.668 | 0.351 | 0.675 | NA |
|  | ET | 0.778 | 0.755 | 0.801 | 0.558 | 0.849 | 50 |
|  | LGBM | 0.771 | 0.738 | 0.803 | 0.544 | 0.843 | 20 |
|  | LR | 0.792 | 0.770 | 0.814 | 0.585 | 0.857 | 100 |
|  | MLP | 0.750 | 0.768 | 0.731 | 0.501 | 0.820 | 200 |
|  | NB | 0.660 | 0.338 | 0.983 | 0.420 | 0.834 | NA |
|  | PLS | 0.771 | 0.732 | 0.810 | 0.545 | 0.839 | NA |
|  | RF | 0.778 | 0.732 | 0.824 | 0.561 | 0.845 | 50 |
|  | SVMLN | 0.791 | 0.764 | 0.818 | 0.584 | 0.858 | 4 |
|  | SVMRBF | 0.770 | 0.715 | 0.824 | 0.545 | 0.846 | 1 |
|  | XGB | 0.762 | 0.694 | 0.831 | 0.531 | 0.840 | 50 |
| RSCharge | KNN | 0.684 | 0.671 | 0.697 | 0.372 | 0.684 | NA |
|  | ADA | 0.761 | 0.741 | 0.782 | 0.525 | 0.842 | 20 |
|  | DT | 0.695 | 0.690 | 0.700 | 0.391 | 0.695 | NA |
|  | ET | 0.761 | 0.732 | 0.790 | 0.526 | 0.842 | 200 |
|  | LGBM | 0.769 | 0.759 | 0.778 | 0.540 | 0.840 | 100 |
|  | LR | 0.775 | 0.766 | 0.784 | 0.552 | 0.843 | 10 |
|  | MLP | 0.767 | 0.755 | 0.778 | 0.537 | 0.844 | 20 |
|  | NB | 0.644 | 0.306 | 0.983 | 0.393 | 0.806 | NA |
|  | PLS | 0.760 | 0.724 | 0.797 | 0.524 | 0.834 | NA |
|  | RF | 0.756 | 0.736 | 0.776 | 0.515 | 0.842 | 200 |
|  | SVMLN | 0.783 | 0.772 | 0.793 | 0.567 | 0.842 | 4 |
|  | SVMRBF | 0.771 | 0.757 | 0.784 | 0.545 | 0.841 | 4 |
|  | XGB | 0.757 | 0.713 | 0.801 | 0.517 | 0.846 | 50 |
| RSDHP | KNN | 0.643 | 0.639 | 0.647 | 0.287 | 0.643 | NA |
|  | ADA | 0.762 | 0.732 | 0.793 | 0.527 | 0.839 | 50 |
|  | DT | 0.699 | 0.694 | 0.704 | 0.400 | 0.699 | NA |
|  | ET | 0.778 | 0.734 | 0.822 | 0.560 | 0.846 | 200 |
|  | LGBM | 0.784 | 0.783 | 0.784 | 0.568 | 0.855 | 200 |
|  | LR | 0.783 | 0.751 | 0.814 | 0.567 | 0.849 | 100 |
|  | MLP | 0.761 | 0.785 | 0.738 | 0.524 | 0.829 | 50 |
|  | NB | 0.635 | 0.287 | 0.983 | 0.375 | 0.828 | NA |
|  | PLS | 0.754 | 0.709 | 0.799 | 0.512 | 0.825 | NA |
|  | RF | 0.776 | 0.741 | 0.812 | 0.555 | 0.853 | 100 |
|  | SVMLN | 0.783 | 0.747 | 0.818 | 0.567 | 0.849 | 16 |
|  | SVMRBF | 0.762 | 0.747 | 0.778 | 0.527 | 0.831 | 2 |
|  | XGB | 0.775 | 0.751 | 0.799 | 0.553 | 0.843 | 200 |
| RSPolar | KNN | 0.701 | 0.698 | 0.704 | 0.404 | 0.701 | NA |
|  | ADA | 0.766 | 0.736 | 0.795 | 0.533 | 0.841 | 50 |
|  | DT | 0.720 | 0.700 | 0.740 | 0.443 | 0.722 | NA |
|  | ET | 0.795 | 0.766 | 0.824 | 0.593 | 0.858 | 100 |
|  | LGBM | 0.773 | 0.753 | 0.793 | 0.549 | 0.859 | 100 |
|  | LR | 0.783 | 0.760 | 0.805 | 0.567 | 0.864 | 100 |
|  | MLP | 0.790 | 0.800 | 0.780 | 0.583 | 0.869 | 100 |
|  | NB | 0.648 | 0.320 | 0.977 | 0.395 | 0.836 | NA |
|  | PLS | 0.764 | 0.709 | 0.818 | 0.532 | 0.834 | NA |
|  | RF | 0.784 | 0.749 | 0.818 | 0.571 | 0.862 | 100 |
|  | SVMLN | 0.790 | 0.766 | 0.814 | 0.582 | 0.863 | 8 |
|  | SVMRBF | 0.776 | 0.787 | 0.765 | 0.554 | 0.855 | 8 |
|  | XGB | 0.779 | 0.762 | 0.797 | 0.561 | 0.859 | 200 |
| RSSecond | KNN | 0.636 | 0.607 | 0.664 | 0.273 | 0.636 | NA |
|  | ADA | 0.735 | 0.702 | 0.767 | 0.472 | 0.787 | 20 |
|  | DT | 0.657 | 0.675 | 0.639 | 0.316 | 0.657 | NA |
|  | ET | 0.766 | 0.743 | 0.789 | 0.534 | 0.848 | 200 |
|  | LGBM | 0.759 | 0.738 | 0.780 | 0.520 | 0.829 | 100 |
|  | LR | 0.751 | 0.713 | 0.789 | 0.505 | 0.817 | 100 |
|  | MLP | 0.736 | 0.747 | 0.725 | 0.473 | 0.816 | 200 |
|  | NB | 0.633 | 0.287 | 0.979 | 0.368 | 0.794 | NA |
|  | PLS | 0.749 | 0.681 | 0.816 | 0.504 | 0.807 | NA |
|  | RF | 0.759 | 0.740 | 0.778 | 0.522 | 0.834 | 100 |
|  | SVMLN | 0.750 | 0.707 | 0.793 | 0.504 | 0.814 | 8 |
|  | SVMRBF | 0.734 | 0.730 | 0.738 | 0.469 | 0.811 | 4 |
|  | XGB | 0.749 | 0.700 | 0.797 | 0.503 | 0.828 | 50 |

**Table S3** Independent test results of 156 baseline models as developed with 13 ML algorithms and 12 feature encoding schemes.

| **Descriptor** | **Method** | **ACC** | **Sn** | **Sp** | **MCC** | **AUC** |
| --- | --- | --- | --- | --- | --- | --- |
| AAC | KNN | 0.717 | 0.737 | 0.697 | 0.435 | 0.717 |
|  | ADA | 0.747 | 0.703 | 0.790 | 0.495 | 0.824 |
|  | DT | 0.646 | 0.644 | 0.647 | 0.291 | 0.646 |
|  | ET | 0.789 | 0.746 | 0.832 | 0.580 | 0.842 |
|  | LGBM | 0.793 | 0.729 | 0.857 | 0.591 | 0.838 |
|  | LR | 0.751 | 0.703 | 0.798 | 0.504 | 0.838 |
|  | MLP | 0.772 | 0.737 | 0.807 | 0.545 | 0.847 |
|  | NB | 0.658 | 0.322 | 0.992 | 0.423 | 0.797 |
|  | PLS | 0.755 | 0.695 | 0.815 | 0.514 | 0.836 |
|  | RF | 0.759 | 0.746 | 0.773 | 0.519 | 0.840 |
|  | SVMLN | 0.738 | 0.669 | 0.807 | 0.481 | 0.834 |
|  | SVMRBF | 0.781 | 0.729 | 0.832 | 0.564 | 0.844 |
|  | XGB | 0.764 | 0.669 | 0.857 | 0.536 | 0.854 |
| AAI | KNN | 0.730 | 0.669 | 0.790 | 0.463 | 0.730 |
|  | ADA | 0.759 | 0.712 | 0.807 | 0.521 | 0.819 |
|  | DT | 0.692 | 0.644 | 0.739 | 0.385 | 0.691 |
|  | ET | 0.755 | 0.712 | 0.798 | 0.512 | 0.857 |
|  | LGBM | 0.793 | 0.763 | 0.824 | 0.587 | 0.864 |
|  | LR | 0.747 | 0.695 | 0.798 | 0.496 | 0.839 |
|  | MLP | 0.768 | 0.720 | 0.815 | 0.538 | 0.849 |
|  | NB | 0.679 | 0.559 | 0.798 | 0.368 | 0.753 |
|  | PLS | 0.722 | 0.703 | 0.739 | 0.443 | 0.806 |
|  | RF | 0.772 | 0.729 | 0.815 | 0.546 | 0.851 |
|  | SVMLN | 0.730 | 0.669 | 0.790 | 0.463 | 0.833 |
|  | SVMRBF | 0.797 | 0.729 | 0.866 | 0.600 | 0.853 |
|  | XGB | 0.785 | 0.746 | 0.824 | 0.571 | 0.856 |
| APAAC | KNN | 0.734 | 0.754 | 0.714 | 0.469 | 0.734 |
|  | ADA | 0.743 | 0.669 | 0.815 | 0.490 | 0.822 |
|  | DT | 0.667 | 0.661 | 0.672 | 0.333 | 0.667 |
|  | ET | 0.806 | 0.737 | 0.874 | 0.617 | 0.861 |
|  | LGBM | 0.814 | 0.771 | 0.857 | 0.631 | 0.834 |
|  | LR | 0.751 | 0.703 | 0.798 | 0.504 | 0.842 |
|  | MLP | 0.751 | 0.746 | 0.756 | 0.502 | 0.825 |
|  | NB | 0.662 | 0.331 | 0.992 | 0.430 | 0.804 |
|  | PLS | 0.755 | 0.695 | 0.815 | 0.514 | 0.837 |
|  | RF | 0.759 | 0.729 | 0.790 | 0.520 | 0.832 |
|  | SVMLN | 0.755 | 0.703 | 0.807 | 0.513 | 0.840 |
|  | SVMRBF | 0.772 | 0.720 | 0.824 | 0.547 | 0.849 |
|  | XGB | 0.734 | 0.636 | 0.832 | 0.477 | 0.805 |
| CTD | KNN | 0.743 | 0.720 | 0.765 | 0.486 | 0.743 |
|  | ADA | 0.827 | 0.822 | 0.832 | 0.654 | 0.918 |
|  | DT | 0.819 | 0.822 | 0.815 | 0.637 | 0.822 |
|  | ET | 0.869 | 0.881 | 0.857 | 0.739 | 0.945 |
|  | LGBM | 0.899 | 0.898 | 0.899 | 0.797 | 0.951 |
|  | LR | 0.806 | 0.805 | 0.807 | 0.612 | 0.902 |
|  | MLP | 0.840 | 0.864 | 0.815 | 0.680 | 0.911 |
|  | NB | 0.759 | 0.729 | 0.790 | 0.520 | 0.848 |
|  | PLS | 0.785 | 0.797 | 0.773 | 0.570 | 0.879 |
|  | RF | 0.895 | 0.949 | 0.840 | 0.794 | 0.942 |
|  | SVMLN | 0.789 | 0.780 | 0.798 | 0.578 | 0.885 |
|  | SVMRBF | 0.835 | 0.847 | 0.824 | 0.671 | 0.913 |
|  | XGB | 0.903 | 0.915 | 0.891 | 0.806 | 0.946 |
| DPC | KNN | 0.713 | 0.780 | 0.647 | 0.430 | 0.713 |
|  | ADA | 0.696 | 0.653 | 0.739 | 0.394 | 0.772 |
|  | DT | 0.684 | 0.653 | 0.714 | 0.368 | 0.683 |
|  | ET | 0.755 | 0.695 | 0.815 | 0.514 | 0.826 |
|  | LGBM | 0.747 | 0.720 | 0.773 | 0.494 | 0.790 |
|  | LR | 0.722 | 0.669 | 0.773 | 0.445 | 0.813 |
|  | MLP | 0.726 | 0.661 | 0.790 | 0.455 | 0.793 |
|  | NB | 0.608 | 0.305 | 0.908 | 0.267 | 0.606 |
|  | PLS | 0.696 | 0.653 | 0.739 | 0.394 | 0.795 |
|  | RF | 0.734 | 0.678 | 0.790 | 0.471 | 0.833 |
|  | SVMLN | 0.709 | 0.644 | 0.773 | 0.421 | 0.762 |
|  | SVMRBF | 0.751 | 0.686 | 0.815 | 0.506 | 0.845 |
|  | XGB | 0.726 | 0.729 | 0.723 | 0.452 | 0.794 |
| PAAC | KNN | 0.743 | 0.754 | 0.731 | 0.485 | 0.743 |
|  | ADA | 0.764 | 0.695 | 0.832 | 0.532 | 0.841 |
|  | DT | 0.662 | 0.610 | 0.714 | 0.326 | 0.661 |
|  | ET | 0.814 | 0.746 | 0.882 | 0.634 | 0.860 |
|  | LGBM | 0.772 | 0.729 | 0.815 | 0.546 | 0.854 |
|  | LR | 0.751 | 0.703 | 0.798 | 0.504 | 0.839 |
|  | MLP | 0.764 | 0.737 | 0.790 | 0.528 | 0.840 |
|  | NB | 0.662 | 0.331 | 0.992 | 0.430 | 0.795 |
|  | PLS | 0.755 | 0.695 | 0.815 | 0.514 | 0.835 |
|  | RF | 0.806 | 0.754 | 0.857 | 0.615 | 0.867 |
|  | SVMLN | 0.747 | 0.695 | 0.798 | 0.496 | 0.835 |
|  | SVMRBF | 0.789 | 0.737 | 0.840 | 0.581 | 0.854 |
|  | XGB | 0.759 | 0.686 | 0.832 | 0.524 | 0.845 |
| PCP | KNN | 0.658 | 0.653 | 0.664 | 0.316 | 0.658 |
|  | ADA | 0.730 | 0.627 | 0.832 | 0.469 | 0.822 |
|  | DT | 0.675 | 0.712 | 0.639 | 0.351 | 0.681 |
|  | ET | 0.730 | 0.678 | 0.782 | 0.462 | 0.794 |
|  | LGBM | 0.764 | 0.729 | 0.798 | 0.528 | 0.825 |
|  | LR | 0.722 | 0.695 | 0.748 | 0.443 | 0.788 |
|  | MLP | 0.759 | 0.653 | 0.866 | 0.531 | 0.817 |
|  | NB | 0.700 | 0.636 | 0.765 | 0.404 | 0.786 |
|  | PLS | 0.662 | 0.627 | 0.697 | 0.325 | 0.763 |
|  | RF | 0.734 | 0.703 | 0.765 | 0.469 | 0.809 |
|  | SVMLN | 0.747 | 0.653 | 0.840 | 0.502 | 0.793 |
|  | SVMRBF | 0.738 | 0.636 | 0.840 | 0.486 | 0.804 |
|  | XGB | 0.738 | 0.619 | 0.857 | 0.490 | 0.809 |
| RSAcid | KNN | 0.730 | 0.712 | 0.748 | 0.460 | 0.730 |
|  | ADA | 0.768 | 0.695 | 0.840 | 0.541 | 0.851 |
|  | DT | 0.684 | 0.737 | 0.630 | 0.370 | 0.684 |
|  | ET | 0.806 | 0.754 | 0.857 | 0.615 | 0.872 |
|  | LGBM | 0.802 | 0.746 | 0.857 | 0.607 | 0.859 |
|  | LR | 0.797 | 0.737 | 0.857 | 0.599 | 0.877 |
|  | MLP | 0.802 | 0.788 | 0.815 | 0.604 | 0.860 |
|  | NB | 0.667 | 0.347 | 0.983 | 0.429 | 0.839 |
|  | PLS | 0.802 | 0.754 | 0.849 | 0.606 | 0.855 |
|  | RF | 0.814 | 0.754 | 0.874 | 0.633 | 0.874 |
|  | SVMLN | 0.802 | 0.763 | 0.840 | 0.605 | 0.871 |
|  | SVMRBF | 0.789 | 0.720 | 0.857 | 0.583 | 0.871 |
|  | XGB | 0.789 | 0.686 | 0.891 | 0.590 | 0.864 |
| RSCharge | KNN | 0.747 | 0.720 | 0.773 | 0.494 | 0.747 |
|  | ADA | 0.772 | 0.720 | 0.824 | 0.547 | 0.840 |
|  | DT | 0.734 | 0.695 | 0.773 | 0.470 | 0.734 |
|  | ET | 0.802 | 0.746 | 0.857 | 0.607 | 0.879 |
|  | LGBM | 0.802 | 0.763 | 0.840 | 0.605 | 0.875 |
|  | LR | 0.802 | 0.754 | 0.849 | 0.606 | 0.867 |
|  | MLP | 0.776 | 0.771 | 0.782 | 0.553 | 0.862 |
|  | NB | 0.654 | 0.322 | 0.983 | 0.407 | 0.820 |
|  | PLS | 0.755 | 0.703 | 0.807 | 0.513 | 0.847 |
|  | RF | 0.823 | 0.780 | 0.866 | 0.648 | 0.881 |
|  | SVMLN | 0.810 | 0.788 | 0.832 | 0.621 | 0.868 |
|  | SVMRBF | 0.810 | 0.771 | 0.849 | 0.622 | 0.875 |
|  | XGB | 0.776 | 0.678 | 0.874 | 0.563 | 0.875 |
| RSDHP | KNN | 0.650 | 0.712 | 0.588 | 0.302 | 0.650 |
|  | ADA | 0.802 | 0.746 | 0.857 | 0.607 | 0.868 |
|  | DT | 0.759 | 0.754 | 0.765 | 0.519 | 0.763 |
|  | ET | 0.806 | 0.720 | 0.891 | 0.620 | 0.864 |
|  | LGBM | 0.806 | 0.754 | 0.857 | 0.615 | 0.885 |
|  | LR | 0.785 | 0.737 | 0.832 | 0.572 | 0.877 |
|  | MLP | 0.764 | 0.746 | 0.782 | 0.528 | 0.858 |
|  | NB | 0.637 | 0.288 | 0.983 | 0.378 | 0.815 |
|  | PLS | 0.785 | 0.746 | 0.824 | 0.571 | 0.839 |
|  | RF | 0.802 | 0.712 | 0.891 | 0.613 | 0.870 |
|  | SVMLN | 0.785 | 0.763 | 0.807 | 0.570 | 0.878 |
|  | SVMRBF | 0.785 | 0.729 | 0.840 | 0.573 | 0.872 |
|  | XGB | 0.810 | 0.797 | 0.824 | 0.620 | 0.875 |
| RSPolar | KNN | 0.726 | 0.712 | 0.739 | 0.452 | 0.726 |
|  | ADA | 0.785 | 0.720 | 0.849 | 0.574 | 0.870 |
|  | DT | 0.738 | 0.754 | 0.723 | 0.477 | 0.738 |
|  | ET | 0.797 | 0.754 | 0.840 | 0.597 | 0.872 |
|  | LGBM | 0.823 | 0.771 | 0.874 | 0.649 | 0.900 |
|  | LR | 0.810 | 0.780 | 0.840 | 0.621 | 0.900 |
|  | MLP | 0.802 | 0.788 | 0.815 | 0.604 | 0.881 |
|  | NB | 0.675 | 0.347 | 1.000 | 0.459 | 0.835 |
|  | PLS | 0.797 | 0.737 | 0.857 | 0.599 | 0.862 |
|  | RF | 0.806 | 0.754 | 0.857 | 0.615 | 0.894 |
|  | SVMLN | 0.789 | 0.763 | 0.815 | 0.579 | 0.894 |
|  | SVMRBF | 0.785 | 0.771 | 0.798 | 0.570 | 0.871 |
|  | XGB | 0.810 | 0.763 | 0.857 | 0.623 | 0.896 |
| RSSecond | KNN | 0.675 | 0.636 | 0.714 | 0.351 | 0.675 |
|  | ADA | 0.751 | 0.720 | 0.782 | 0.503 | 0.834 |
|  | DT | 0.709 | 0.712 | 0.706 | 0.418 | 0.709 |
|  | ET | 0.806 | 0.771 | 0.840 | 0.613 | 0.872 |
|  | LGBM | 0.785 | 0.754 | 0.815 | 0.571 | 0.871 |
|  | LR | 0.755 | 0.686 | 0.824 | 0.515 | 0.832 |
|  | MLP | 0.751 | 0.729 | 0.773 | 0.502 | 0.847 |
|  | NB | 0.646 | 0.288 | 1.000 | 0.411 | 0.819 |
|  | PLS | 0.755 | 0.686 | 0.824 | 0.515 | 0.827 |
|  | RF | 0.789 | 0.771 | 0.807 | 0.578 | 0.858 |
|  | SVMLN | 0.738 | 0.669 | 0.807 | 0.481 | 0.830 |
|  | SVMRBF | 0.785 | 0.746 | 0.824 | 0.571 | 0.861 |
|  | XGB | 0.785 | 0.720 | 0.849 | 0.574 | 0.859 |

**Table S4.** Detailed prediction results of TAP 1.0, iTTCA-Hybrid, iTTCA-RF, PSATTCA, and StackTTCA on case studies

| **#** | **True** | **TAP 1.0** | **iTTCA-Hybrid** | **iTTCA-RF** | **PSATTCA** | **StackTTCA** |
| --- | --- | --- | --- | --- | --- | --- |
| 1 | TTCA | TTCA | TTCA | TTCA | TTCA | TTCA |
| 2 | TTCA | TTCA | non-TTCA | TTCA | TTCA | TTCA |
| 3 | TTCA | TTCA | TTCA | TTCA | TTCA | non-TTCA |
| 4 | TTCA | non-TTCA | TTCA | TTCA | TTCA | TTCA |
| 5 | TTCA | TTCA | TTCA | non-TTCA | TTCA | TTCA |
| 6 | TTCA | TTCA | TTCA | TTCA | TTCA | TTCA |
| 7 | TTCA | TTCA | TTCA | non-TTCA | non-TTCA | TTCA |
| 8 | TTCA | TTCA | TTCA | TTCA | non-TTCA | TTCA |
| 9 | TTCA | TTCA | TTCA | non-TTCA | TTCA | TTCA |
| 10 | TTCA | TTCA | TTCA | non-TTCA | non-TTCA | non-TTCA |
| 11 | TTCA | non-TTCA | TTCA | TTCA | TTCA | TTCA |
| 12 | TTCA | TTCA | TTCA | TTCA | TTCA | TTCA |
| 13 | TTCA | TTCA | TTCA | non-TTCA | TTCA | TTCA |
| 14 | TTCA | non-TTCA | non-TTCA | non-TTCA | TTCA | TTCA |
| 15 | TTCA | non-TTCA | TTCA | TTCA | TTCA | TTCA |
| 16 | TTCA | TTCA | TTCA | TTCA | TTCA | TTCA |
| 17 | TTCA | TTCA | TTCA | TTCA | non-TTCA | TTCA |
| 18 | TTCA | TTCA | non-TTCA | TTCA | TTCA | TTCA |
| 19 | TTCA | non-TTCA | TTCA | TTCA | TTCA | TTCA |
| 20 | TTCA | non-TTCA | TTCA | TTCA | non-TTCA | TTCA |
| 21 | TTCA | TTCA | TTCA | TTCA | TTCA | TTCA |
| 22 | TTCA | TTCA | TTCA | TTCA | TTCA | TTCA |
| 23 | TTCA | non-TTCA | non-TTCA | TTCA | TTCA | TTCA |
| 24 | TTCA | TTCA | TTCA | TTCA | non-TTCA | TTCA |
| 25 | TTCA | TTCA | TTCA | non-TTCA | TTCA | TTCA |
| 26 | TTCA | TTCA | TTCA | TTCA | TTCA | TTCA |
| 27 | TTCA | TTCA | TTCA | TTCA | non-TTCA | TTCA |
| 28 | TTCA | TTCA | TTCA | TTCA | TTCA | TTCA |
| 29 | TTCA | TTCA | non-TTCA | non-TTCA | non-TTCA | TTCA |
| 30 | TTCA | TTCA | TTCA | TTCA | non-TTCA | non-TTCA |
| 31 | TTCA | non-TTCA | TTCA | TTCA | non-TTCA | TTCA |
| 32 | TTCA | non-TTCA | TTCA | TTCA | non-TTCA | TTCA |
| 33 | TTCA | TTCA | TTCA | TTCA | non-TTCA | TTCA |
| 34 | TTCA | TTCA | TTCA | TTCA | TTCA | TTCA |
| 35 | TTCA | TTCA | TTCA | TTCA | TTCA | TTCA |
| 36 | TTCA | TTCA | TTCA | TTCA | TTCA | TTCA |
| 37 | TTCA | non-TTCA | TTCA | TTCA | non-TTCA | non-TTCA |
| 38 | TTCA | non-TTCA | TTCA | TTCA | non-TTCA | non-TTCA |
| 39 | TTCA | TTCA | TTCA | TTCA | TTCA | TTCA |
| 40 | TTCA | non-TTCA | TTCA | TTCA | TTCA | TTCA |
| 41 | TTCA | non-TTCA | TTCA | TTCA | TTCA | TTCA |
| 42 | TTCA | TTCA | TTCA | TTCA | TTCA | TTCA |
| 43 | TTCA | non-TTCA | TTCA | TTCA | TTCA | TTCA |
| 44 | TTCA | TTCA | TTCA | TTCA | TTCA | TTCA |
| 45 | TTCA | non-TTCA | non-TTCA | TTCA | non-TTCA | TTCA |
| 46 | TTCA | TTCA | non-TTCA | TTCA | TTCA | TTCA |
| 47 | TTCA | TTCA | TTCA | TTCA | TTCA | non-TTCA |
| 48 | TTCA | non-TTCA | TTCA | TTCA | TTCA | non-TTCA |
| 49 | TTCA | non-TTCA | non-TTCA | TTCA | TTCA | non-TTCA |
| 50 | TTCA | non-TTCA | TTCA | non-TTCA | TTCA | TTCA |
| 51 | TTCA | non-TTCA | TTCA | TTCA | non-TTCA | TTCA |
| 52 | TTCA | non-TTCA | TTCA | TTCA | TTCA | non-TTCA |
| 53 | TTCA | TTCA | TTCA | non-TTCA | TTCA | TTCA |
| 54 | TTCA | TTCA | TTCA | TTCA | TTCA | TTCA |
| 55 | TTCA | TTCA | non-TTCA | non-TTCA | TTCA | TTCA |
| 56 | TTCA | TTCA | TTCA | non-TTCA | TTCA | TTCA |
| 57 | TTCA | non-TTCA | non-TTCA | TTCA | non-TTCA | non-TTCA |
| 58 | TTCA | TTCA | non-TTCA | TTCA | TTCA | TTCA |
| 59 | TTCA | TTCA | TTCA | non-TTCA | TTCA | TTCA |
| 60 | TTCA | TTCA | TTCA | non-TTCA | TTCA | TTCA |
| 61 | TTCA | TTCA | TTCA | TTCA | TTCA | TTCA |
| 62 | TTCA | non-TTCA | TTCA | TTCA | TTCA | TTCA |
| 63 | TTCA | non-TTCA | non-TTCA | TTCA | TTCA | TTCA |
| 64 | TTCA | TTCA | non-TTCA | TTCA | TTCA | TTCA |
| 65 | TTCA | non-TTCA | non-TTCA | TTCA | TTCA | non-TTCA |
| 66 | TTCA | non-TTCA | non-TTCA | non-TTCA | TTCA | TTCA |
| 67 | TTCA | TTCA | non-TTCA | non-TTCA | TTCA | TTCA |
| 68 | TTCA | TTCA | TTCA | non-TTCA | TTCA | non-TTCA |
| 69 | TTCA | non-TTCA | non-TTCA | non-TTCA | non-TTCA | non-TTCA |
| 70 | TTCA | TTCA | non-TTCA | TTCA | TTCA | TTCA |
| 71 | TTCA | TTCA | non-TTCA | non-TTCA | TTCA | TTCA |
| 72 | TTCA | non-TTCA | non-TTCA | non-TTCA | TTCA | TTCA |
| 73 | TTCA | non-TTCA | non-TTCA | non-TTCA | TTCA | TTCA |

Note that prediction results of iTTCA-Hybrid, TAP1.0, iTTCA-RF, and PSRTTCA were obtained the study of PSRTTCA [1].

## Reference

[1] P. Charoenkwan, C. Pipattanaboon, C. Nantasenamat, M. M. Hasan, M. A. Moni, and W. Shoombuatong, "PSRTTCA: A new approach for improving the prediction and characterization of tumor T cell antigens using propensity score representation learning," *Computers in Biology and Medicine,* vol. 152, p. 106368, 2023.
